# Men who have sex with men in Great Britain: comparing methods and estimates from probability and convenience sample surveys

**DOI:** 10.1136/sextrans-2015-052389

**Published:** 2016-03-10

**Authors:** Philip Prah, Ford Hickson, Chris Bonell, Lisa M McDaid, Anne M Johnson, Sonali Wayal, Soazig Clifton, Pam Sonnenberg, Anthony Nardone, Bob Erens, Andrew J Copas, Julie Riddell, Peter Weatherburn, Catherine H Mercer

**Affiliations:** 1 Research Department of Infection & Population Health, University College London, London, UK; 2 Sigma Research, London School of Hygiene & Tropical Medicine, London, UK; 3 UCL Institute of Education, London, UK; 4 MRC/CSO Social & Public Health Sciences Unit, University of Glasgow, Glasgow, UK; 5 HIV/STI Department, Public Health England, London, UK; 6 Department of Health Services Research and Policy, London School of Hygiene & Tropical Medicine, London, UK; 7 Department of Social & Environmental Health Research, London School of Hygiene & Tropical Medicine, London, UK

**Keywords:** GAY MEN, SEXUAL BEHAVIOUR, SEXUAL HEALTH

## Abstract

**Objective:**

To examine sociodemographic and behavioural differences between men who have sex with men (MSM) participating in recent UK convenience surveys and a national probability sample survey.

**Methods:**

We compared 148 MSM aged 18–64 years interviewed for Britain's third National Survey of Sexual Attitudes and Lifestyles (Natsal-3) undertaken in 2010–2012, with men in the same age range participating in contemporaneous convenience surveys of MSM: 15 500 British resident men in the European MSM Internet Survey (EMIS); 797 in the London Gay Men's Sexual Health Survey; and 1234 in Scotland's Gay Men's Sexual Health Survey. Analyses compared men reporting at least one male sexual partner (past year) on similarly worded questions and multivariable analyses accounted for sociodemographic differences between the surveys.

**Results:**

MSM in convenience surveys were younger and better educated than MSM in Natsal-3, and a larger proportion identified as gay (85%–95% vs 62%). Partner numbers were higher and same-sex anal sex more common in convenience surveys. Unprotected anal intercourse was more commonly reported in EMIS. Compared with Natsal-3, MSM in convenience surveys were more likely to report gonorrhoea diagnoses and HIV testing (both past year). Differences between the samples were reduced when restricting analysis to gay-identifying MSM.

**Conclusions:**

National probability surveys better reflect the population of MSM but are limited by their smaller samples of MSM. Convenience surveys recruit larger samples of MSM but tend to over-represent MSM identifying as gay and reporting more sexual risk behaviours. Because both sampling strategies have strengths and weaknesses, methods are needed to triangulate data from probability and convenience surveys.

## Introduction

The emergence of the HIV epidemic in the 1980s prompted an unprecedented medical and social science interest in the sexual behaviour of men who have sex with men (MSM). Currently in Britain, an estimated 3% of men aged 16–74 years report sex with one or more men in the past 5 years.^1^ In 2013, 61% of HIV infections acquired in the UK were among MSM.^2^ ^3^

To inform health promotion for this population in the UK, various surveys have been undertaken. Currently, all large surveys of MSM in the UK recruit using convenience sampling. Convenience surveys have traditionally been venue-based such as at Gay Pride events, or in gay bars and clubs in the case of the London Gay Men's Sexual Health Survey (London-GMSHS) (S Wayal *et al*. Temporal trends in HIV testing and undiagnosed HIV in community sample of men who have sex with men in London, UK 2000–13: an observational study. *Lancet HIV* 2015 (in review).) and Scotland's Gay Men's Sexual Health Survey (Scotland-GMSHS).^4^ More recently, web-based convenience surveys are being used, such as the European MSM Internet Survey (EMIS), which includes British men.^5^ These surveys recruit certain subgroups of MSM but the proportion and particularities of the MSM population represented in such surveys is unknown. Probability sample surveys like Britain's National Survey of Sexual Attitudes and Lifestyles (Natsal) should recruit a more representative sample of MSM. However, because the proportion of men having sex with men is relatively low,^1^ the sample of MSM in general population surveys like Natsal is small, precluding anything but relatively rudimentary analyses. Nonetheless, Natsal enables assessment of the proportion of MSM who attend gay bars and clubs or use the internet to find a sexual partner, which are potentially useful in assessing the selection biases inherent in convenience surveys of MSM recruited through venues and websites.

Previous research has shown that MSM participating in venue-based convenience surveys were more likely to be younger, report greater sexual risk behaviours and sexually transmitted infection (STI) diagnoses than MSM who participated in Natsal-2.^6^ ^7^ This is consistent with some international comparisons, which found greater risk behaviour reported by MSM in convenience surveys.^8^ However, some international studies have suggested that some convenience surveys provide similar estimates of sexual risk behaviours as probability surveys.^9^ Because the recruitment methods used by venue-based and web-based convenience surveys change as do MSM's use of venues and websites, regular comparisons between convenience surveys and probability sample surveys are needed.

This study is the first to compare several convenience surveys of MSM in Britain carried out in 2010–2012 with a population probability survey (Natsal-3) carried out contemporaneously. We begin by drawing on Natsal-3 data to calculate the proportion of MSM who use gay venues and seek a sexual partner on the internet. We then compare the three convenience surveys with Natsal-3 to calculate differences in sociodemographic characteristics, drug use, sexual behaviour and sexual health characteristics. In addition, we investigate the influence of gay identity on the observed differences.

## Methods

This paper compares data from a national probability sample survey, Natsal-3, with data from three surveys that used convenience sampling: EMIS London-GMSHS and Scotland-GMSHS. To be included in these analyses, research participants were required to have reported at least one male sexual partner in the year prior to data collection, being resident in Britain and being aged 18–64 years. Our analyses involved comparison between the surveys where questions on sociodemographics, drug use, sexual behaviour and sexual health had similar wordings (table 1). Further details of each survey are reported below.

**Table 1:** Question wording used in the different surveys

	Natsal-3	EMIS	London-GMSHS	Scotland-GMSHS
Demographics				
Age	What was your age last birthday?	How old are you?	What was your age at your last birthday? Years	What age are you?
Academic qualification	Please read down the list and tell me the highest qualification that you have	What is your highest education qualification?		Could you tell us what your highest educational qualifications are?
Employment	Which of these descriptions applies to what you were doing last week, that is, in the 7 days ending last Sunday?	Which of the following best describes your current occupation?	Are you employed at present? Yes/no	Are you currently employed/self-employed/unemployed retired/student?
Ethnicity	To which of the ethnic groups on this card do you consider you belong? White/mixed, Asian or Asian British/black or British black/Chinese or other		Which of the following ethnic groups best describes you? White/black/SE Asian/Asian/mixed/other	
Urban	Urban/rural indicator classification. Urban ≥10k*	How would you describe the place you live in? 1=a million or more 2=500 000–999 999 3=100 000–499 999 4=10 000–99 999 5=less than 10 000 people		
Region: Country	Region*	Which country do you currently live in?		
Sexual identity	Which of the options on this card best describes how you think of yourself? Straight/gay/bisexual/other	Which of the following options best describes how you think of yourself? Gay/bisexual/straight/other/I don't use a term	How would you describe your sexual orientation? Gay/bisexual/straight/other	How would you describe your sexual orientation? Gay/bisexual/straight/other
Attraction scale	I have felt sexually attracted… 1=Only to men 2=Mostly to men and sometimes to women 3=Both to men and women equally 4=Mostly to women and sometimes to men 5=Only to women	Who are you sexually attracted to? 1=Only to men 2=Mostly to men and sometimes to women 3=Both to men and women equally 4=Mostly to women and sometimes to men 5=Only to women		
Sexual partnerships				
First same-sex experience before age 16	And how old were you the first time you had sex with a (man) involving (genital area/penis) contact?	How old were you the very first time you had any kind of sex with a man/boy, or a man/boy had any kind of sex with you?		
Number of male sexual partners, past year	In the last year how many men have you had sex with?	How many different steady/non-steady male partners have you had sex with in the last 12 months?	In the last year, with how many men have you had sex?	With how many men have you had any sexual contact in the last 12 months?
One or more female sexual partners, past year	Altogether, in your last year, how many women have you had sexual intercourse with? (Sexual intercourse was previously defined in the questionnaire as including vaginal, anal and oral sex)	When did you last have any kind of sex with a woman? (Sex was previously defined to mean physical contact to orgasm (close to orgasm)		
Sexual practices				
Same-sex anal sex, past year	When, if ever, was the last occasion you had anal sex with a man—by you to him?/by him to you?	When did you last have anal intercourse with a man (either with or without a condom)	In the last year, with how many men have you had active/passive anal intercourse?	With how many men have you had anal sex in the last 12 months?
Unprotected anal intercourse, past year	In the last year, with how many men have you had anal intercourse without using a condom?	How many different steady/non-steady male partners have you had anal intercourse without a condom within the last 12 months?	In the last year, with how many men have you had active/passive anal intercourse without a condom?	With how many men have you had anal sex WITHOUT a condom in the last 12 months?
HIV testing and clinic attendance				
HIV test	‘*(Apart from when you were donating blood)* Have you ever had a test for HIV (the virus that causes AIDS)?’ ‘When was that test? (the last HIV test if more than one)’	Have you ever received an HIV test result? If positive: In which year were you first diagnosed HIV positive? If HIV negative: When did you last have an HIV test?	When did you have your last named HIV test (not anonymous)? ≤1/>1–5 years/>5/never	When was your most recent HIV test?
Attended a sexual health clinic, past year	Have you ever attended a sexual health clinic (GUM clinic)? When was that?		Have you attended a sexual health/GUM clinic in the last year?	
STI diagnosis				
Diagnosed with: Gonorrhoea Chlamydia Syphilis	When were you last told by a doctor or healthcare professional that you had… Gonorrhoea Chlamydia Syphilis	Have you ever been diagnosed with …? When were you last diagnosed with…? Gonorrhoea Chlamydia Syphilis	Have you had an STI in the last year? *If yes, which of the following STIs have you had* Gonorrhoea/chlamydia/syphilis/LGV/other	
Drug use				
Ever taken amyl nitrates	Have you ever taken any of the drugs listed below? (Please do not count any drugs you have injected): amyl nitrates	When was the last time you consumed poppers (nitrite inhalants)?		
Recreational drug use	Have you ever taken any of the drugs listed below? (Please do not count any drugs you have injected)	Have you ever taken any other recreational or illicit drugs?		
Injecting drug use	Have you ever injected yourself with any non-prescribed drugs or other substances? When was the last time you injected yourself with non-prescribed drugs or other substances	Have you ever injected any drug other than anabolic steroids or medicines? Never/last 12 months/more than 12 months ago		

*Information provided by the ONS—UK's national statistical institute and the largest producer of official statistics in the UK. The ONS provide standardised classification of area population size and region.^10^

EMIS, European MSM (men who have sex with men) Internet Survey; GMSHS, Gay Men's Sexual Health Survey; Natsal-3, National Survey of Sexual Attitudes and Lifestyles; ONS, Office of national statistics; STI, sexually transmitted infection; GUM, genito-urinary medicine; LGV, lymphogranumola venereum.

### Natsal-3

The Natsal-3 survey used a multistage, stratified random probability sample design.^1^ ^11^ Using addresses from the comprehensive Small User Postcode Address File as the sampling frame, households in Great Britain were selected at random, and one individual aged 16–74 was randomly selected from each household. Individuals aged 16–34 years were oversampled. Data collection occurred between September 2010 and August 2012. Participants were interviewed face-to-face using computer-assisted personal interviewing (CAPI) and computer-assisted self-interviewing (CASI) for the more sensitive topics. Sociodemographics were assessed in the CAPI and drug use, sexual behaviour and sexual health in the CASI. With a response rate of 57.7% (interviews completed from eligible addresses) and a co-operation rate of 65.8% (interviews completed from eligible addresses contacted), Natsal-3 achieved a total sample size of 15 162 participants. A total of 148 MSM met the inclusion criteria for the analyses reported here.

### EMIS

EMIS 2010 was a self-completion online sexual health needs assessment survey.^5^ The survey was promoted on over 230 websites aiming to appeal to gay and other MSM, including Gaydar, Manhunt, Gay Romeo and Terence Higgins Trust, as well as via posters and postcards distributed at gay venues. Conducted across 38 countries in 25 languages, data collection ran from June 2010 to August 2010. Over 180 000 men aged between 18 and 88 years across Europe participated, including 18 435 MSM resident in England, Scotland, Wales and Northern Ireland. MSM from Northern Ireland participants were excluded from the analyses reported here to increase comparability with Natsal-3. A total of 15 500 men met the inclusion criteria for our analyses.

### London-GMSHS

Men attending gay bars, clubs and saunas across London were recruited in 2011 (S Wayal *et al*. *Lancet HIV* 2015 (in review).). Participants were given a self-completion pen-and-paper questionnaire. A total sample of 1185 men, aged between 18 and 81 years, was recruited (response rate 61%). This survey did not record the country of residence of people living outside of London. Therefore, only London residents (797 men) were included in our analyses.

### Scotland-GMSHS

Conducted every 3 years for a 2-week period,^4^ participants were recruited from 15 gay bars and two saunas across Edinburgh and Glasgow. Recruitment occurred at two time-points in the evening each day of the week. All men present at the time of recruitment were approached and asked to self-complete a pen-and-paper questionnaire. In 2011, with a response rate of 65.2%, a total sample of 1515 men, aged between 18 and 83 years, was recruited. The analyses reported here were restricted to a total of 1234 men resident in Scotland.

### Statistical methods

Analyses were conducted using the complex survey functions in Stata 13.1. Natsal-3 data were weighted to account for differential probability of selection and non-response by age, sex and region. For each variable, frequencies are reported for all surveys; 95% CIs are reported only for Natsal-3 since these are not appropriate in the case of prevalence estimates from convenience samples as they were narrow and so contribute little to the comparison between surveys. We first estimated the proportion of MSM in Natsal-3 who reported use of gay venues and seeking sex via the internet. We then compared each convenience survey with Natsal-3 individually on all our variables. First, survey-equivalent χ^2^ tests were used to test for differences in sociodemographic characteristics. Then forward stepwise regression was used to identify the sociodemographic differences associated with participating in each convenience survey compared with Natsal-3. Logistic regression was then used to compare drug use, sexual behaviour and sexual health in the convenience surveys compared with Natsal-3, crude ORs and ORs after adjusting for sociodemographic differences. Finally, we repeated these comparisons, restricting analyses to those MSM identifying as gay. Due to the small sample size in Natsal-3, resulting in insufficient power, we were unable to formally test this as an interaction.

## Results

### Estimating the proportion of MSM using gay venues and seeking sex on the internet

Among MSM in Natsal-3, 52.4% (95% CI 42.1% to 62.4%) reported visiting a gay pub, bar or club at least once in the past year; while 41.4% (95% CI 32.3% to 51.1%) reported using the internet to find a sexual partner in the past year.

### Sociodemographic characteristics

Compared with those participating in Natsal-3, MSM in the convenience surveys tended to be younger and more likely to report education to at least higher education (table 2). MSM in EMIS were more likely to report living in London. No significant differences in employment were found comparing Natsal-3 with EMIS and with Scotland-GMSHS. However, men in the London-GMSHS were more likely to be employed and also to be of non-white ethnicity. With respect to sexual identity, 62.4% (95% CI 52.0% to 71.7%) of MSM in Natsal-3 reported identifying as gay, but this was more commonly reported in all three convenience surveys: 84.7% (EMIS), 94.4% (London-GMSHS) and 90.8% (Scotland-GMSHS).

**Table 2:** Demographic characteristics: convenience surveys relative to Natsal-3

	Natsal-3	EMIS	London-GMSHS	Scotland-GMSHS
Median age (IQR)	41 (27–48)	36 (27–45)	33 (27–40)	30 (24–40)
Age group				
18–24	16.5% (11.2% to 23.7%)	15.9%	13.4%	28.4%
25–34	25.1% (18.5% to 33.0%)	30.5%	42.8%	35.3%
25–44	17.4% (10.5% to 27.4%)	27.1%	30.4%	21.9%
45–64	41.0% (31.8% to 50.9%)	26.5%	13.4%	14.4%
p Value		0.005	<0.001	<0.001
Academic qualifications				
Degree-level qualification	38.7% (29.7% to 48.6%)	47.8%		45.9%
Higher education, A-level or equivalent	18.9% (12.6% to 27.3%)	33.7%		38.1%
GCSE, O-level or equivalent	32.2% (24.2% to 41.3%)	15.9%		14.5%
None	10.2% (5.5% to 18.1%)	2.6%		1.5%
p Value		<0.001		<0.001
Employment				
Employed	70.6% (61.3% to 78.5%)	66.7%	88.0%	70.4%
Other/unemployed	29.4% (21.5% to 38.7%)	33.3%	12.0%	29.6%
p Value		0.396	<0.001	0.902
Ethnicity (binary)				
White	96.3% (89.7% to 98.7%)		83.3%	
Non-white	3.7% (1.3% to 10.3%)		16.7%	
p Value			0.001	
London resident				
No	78.8% (68.1% to 86.6%)	62.1%		
Yes	21.2% (13.4% to 31.9%)	37.9%		
p Value		0.003		
Urban area				
Rural or town area (<10 000)	15.4% (9.3% to 24.4%)	9.6%		
Urban area (>10 000)	84.6% (75.6% to 90.7%)	90.4%		
p Value		0.060		
Country				
England	85.2% (77.0% to 90.8%)	89.6%		
Scotland	7.6% (3.9% to 14.0%)	7.3%		
Wales	7.2% (3.5% to 14.3%)	3.1%		
p Value		0.047		
Sexual identity				
Gay	62.4% (52.0% to 71.7%)	84.7%	94.4%	90.8%
Bisexual	16.8% (10.6% to 25.6%)	10.6%	5.1%	8.4%
Heterosexual	20.8% (13.3% to 30.9%)	0.3%	0.3%	0.2%
p Value		<0.001	<0.001	<0.001
Attraction scale				
Opposite sex only	6.7% (2.8% to 15.1%)	0.0%		
More often opposite sex, and at least once same sex	13.6% (7.8% to 22.7%)	2.3%		
About equally often to opposite sex and same sex	9.5% (5.6% to 15.6%)	3.6%		
More often same sex, and at least once opposite sex	34.8% (26.2% to 44.6%)	16.4%		
Same sex only	35.5% (26.8% to 45.2%)	77.7%		
p Value		<0.001		
Denominator	148, 150	15 500	797	1234

p Values from χ^2^ test compared with Natsal-3.

EMIS, European MSM (men who have sex with men) Internet Survey; GMSHS, Gay Men's Sexual Health Survey; Natsal-3, National Survey of Sexual Attitudes and Lifestyles.

### Drug use

Recreational drug use in the past year (adjusted odds ratio (AOR): 3.62, 95% CI 2.33 to 5.61) and ever having taken amyl nitrates (AOR: 5.21, 95% CI 3.40 to 7.98) were more likely to be reported in EMIS than Natsal-3. No significant difference was found with respect to reporting injecting non-prescribed drugs in the past year.

### Sexual behaviours

An estimated 26.4% (95% CI 18.2% to 36.5%) of MSM in Natsal-3 reported a same-sex sexual experience before age 16 (table 3), similar to that observed in EMIS (28.5%). Reporting at least five male sexual partners in the past year was more common among MSM participating in EMIS (AOR: 4.28, 95% CI 2.63 to 6.97), London-GMSHS (AOR: 4.06, 95% CI 2.40 to 6.88) and Scotland-GMSHS (AOR: 2.23, 95% CI 1.34 to 3.74) than Natsal-3. Conversely, reporting female sexual partner(s) was rarer among MSM participating in EMIS than Natsal-3. Same-sex anal sex in the past year was more likely to be reported among MSM participating in EMIS and London-GMSHS than Natsal-3, while no difference was found between Natsal-3 and Scotland-GMSHS after adjusting for sociodemographic differences. Unprotected anal intercourse (UAI) with multiple partners in the past year was more commonly reported among MSM participating in EMIS than Natsal-3 (AOR: 2.30, 95% CI 1.16 to 4.59) but no differences were found comparing Natsal-3 with London-GMSHS or Scotland-GMSHS.

**Table 3:** Sexual behaviours, HIV testing, reported STI diagnoses and drug use: convenience surveys relative to Natsal-3

	Natsal-3	EMIS	London-GMSHS	Scotland-GMSHS
*Sexual partners*				
First same-sex experience before age 16				
%	26.4 (18.2 to 36.5)	28.5	–	–
Crude OR	1.00	1.12 (0.69 to 1.80)		
AOR	1.00	1.35 (0.84 to 2.18)		
At least five male sexual partners, past year				
%	23.2 (15.5 to 33.1)	57.6	53.8	40.9
Crude OR	1.00	4.51 (2.74 to 7.43)	3.87 (2.31 to 6.48)	2.30 (1.38 to 3.83)
AOR	1.00	4.28 (2.63 to 6.97)	4.06 (2.40 to 6.88)	2.23 (1.34 to 3.74)
One or more female sexual partners, past year				
%	28.0 (19.7 to 38.1)	9.3	–	–
Crude OR	1.00	0.26 (0.17 to 0.42)		
AOR	1.00	0.30 (0.19 to 0.47)		
*Sexual practices*				
Same sex anal sex, past year				
%	73.3 (63.4 to 81.3)	87.4	91.7	85.7
Crude OR	1.00	2.51 (1.58 to 4.00)	4.03 (2.37 to 6.84)	2.14 (1.31 to 3.48)
AOR	1.00	2.36 (1.48 to 3.76)	3.63 (2.09 to 6.30)	1.49 (0.89 to 2.50)
Unprotected anal intercourse (with 2+ partners), past year				
%	13.4 (7.4 to 23.1)	25.2	21.6	14.9
Crude OR	1.00	2.18 (1.12 to 4.23)	1.78 (0.90 to 3.54)	1.06 (0.54 to 2.09)
AOR	1.00	2.30 (1.18 to 4.59)	1.61 (0.79 to 3.28)	0.91 (0.44 to 1.89)
*HIV testing and clinic attendance*				
HIV test, ever				
%	60.1 (48.9 to 70.3)	75.4	91.8	83.2
Crude OR	1.00	2.03 (1.29 to 3.20)	7.43 (4.42 to 12.49)	3.29 (2.04 to 5.31)
AOR	1.00	1.73 (1.07 to 2.80)	7.95 (4.58 to 13.78)	2.99 (1.86 to 4.79)
HIV test, past year				
%	22.2 (15.6 to 30.6)	42.2	58.1	52.8
Crude OR	1.00	2.56 (1.66 to 3.95)	4.85 (3.08 to 7.65)	3.78 (2.42 to 5.91)
AOR	1.00	2.26 (1.47 to 3.47)	4.39 (2.73 to 7.06)	3.01 (1.91 to 4.75)
Attended a sexual health clinic, past year				
%	39.2 (28.0 to 51.6)	–	57.4	–
Crude OR	1.00		2.09 (1.24 to 3.53)	
AOR	1.00		2.04 (1.22 to 3.42)	
*STI diagnoses*				
Diagnosed with an STI, past year*				
%	5.0 (2.3 to 10.5)	9.5	11.6	–
Crude OR	1.00	2.01 (0.90 to 4.51)	2.50 (1.08 to 5.76)	
AOR	1.00	1.91 (0.85 to 4.30)	2.43 (0.99 to 5.99)	
Diagnosed with chlamydia, past year				
%	3.5 (1.4 to 8.5)	5.7	6.4	–
Crude OR	1.00	1.69 (0.65 to 4.39)	1.91 (0.71 to 5.14)	
AOR	1.00	1.66 (0.63 to 4.32)	1.94 (0.63 to 5.92)	
Diagnosed with gonorrhoea, past year				
%	0.5 (0.1 to 3.2)	3.8	6.3	–
Crude OR	1.00	8.47 (1.18 to 60.89)	14.39 (1.96 to 105.47)	
AOR	1.00	8.08 (1.12 to 58.2)	14.36 (1.86 to 110.86)	
Diagnosed with syphilis, past year				
%	1.0 (0.1 to 6.9)	2.3	2.8	–
Crude OR	1.00	2.26 (0.32 to 16.13)	2.71 (0.36 to 20.15)	
AOR	1.00	2.20 (0.32 to 15.18)	3.33 (0.39 to 28.18)	
*Drug use*				
Ever taken amyl nitrates				
%	35.0 (26.4 to 44.7)	72.6	–	–
Crude OR	1.00	4.92 (3.27 to 7.39)		
AOR	1.00	5.21 (3.40 to 7.98)		
Drug use, past year				
%	29.2 (21.1 to 38.9)	60.7	–	–
Crude OR	1.00	3.74 (2.42 to 5.79)		
AOR	1.00	3.62 (2.33 to 5.61)		
Injected non-prescribed drugs, past year				
%	0.5 (0.1 to 3.6)	3.0	–	–
Crude OR	1.00	5.85 (0.81 to 42.08)		
AOR	1.00	5.34 (0.73 to 38.98)		

AOR: adjusted for age, academic qualification and London residency (EMIS); age, employment and ethnicity (London-GMSHS); age and academic qualification (Scotland-GMSHS).

*Reported at least one diagnosis of chlamydia, gonorrhoea and/or syphilis.

EMIS, European MSM (men who have sex with men) Internet Survey; GMSHS, Gay Men's Sexual Health Survey; Natsal-3, National Survey of Sexual Attitudes and Lifestyles; STI, sexually transmitted infection.

### HIV testing

HIV testing in the past year was consistently more commonly reported among MSM participating in the convenience samples than Natsal-3. A similar relationship was found for ever having tested for HIV (table 3).

### STI diagnoses

MSM participating in London-GMSHS were more likely to report attending a sexual health clinic in the past year than those participating in Natsal-3. The reported prevalence of gonorrhoea diagnosis in the past year was 0.5% (95% CI 0.1% to 3.2%) among Natsal-3 MSM, which was significantly lower than that found among MSM participating in EMIS (3.8%) or London-GMSHS (5.9%). No significant differences in prevalence of diagnoses of syphilis or chlamydia were found between surveys.

### MSM who identify as gay

**Figure 1:**
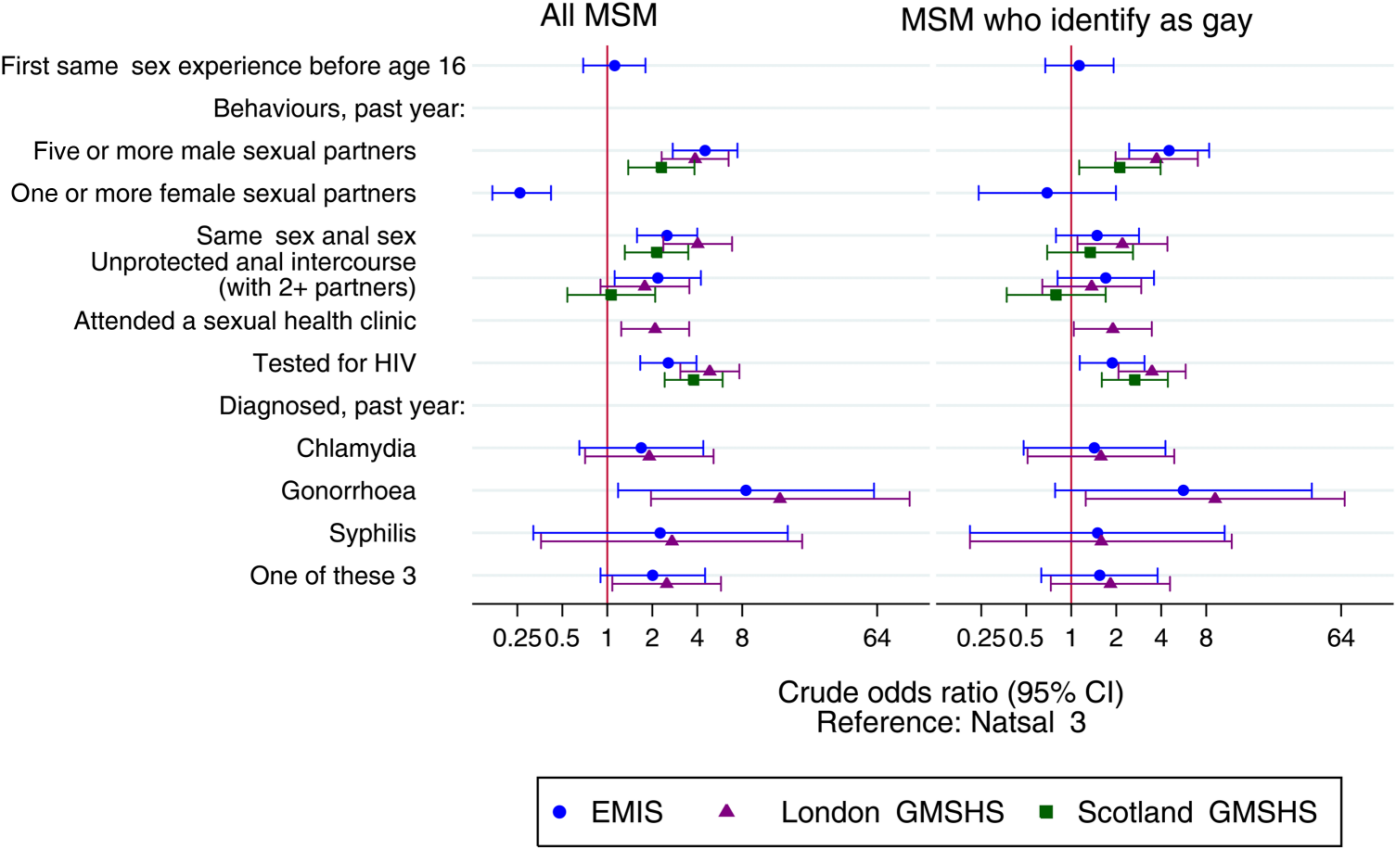
OR (95% CI) for sexually transmitted infection (STI) diagnosis, testing and sexual practices and behaviours in the convenience samples relative to Natsal-3, in men who have sex with men (MSM) who identify as gay. EMIS, European MSM (men who have sex with men) Internet Survey; GMSHS, Gay Men's Sexual Health Survey; MSM, men who have sex with men; Natsal-3, National Survey of Sexual Attitudes and Lifestyles.

We undertook a subgroup analysis of MSM who reported identifying as gay in each survey, corresponding to sample sizes of 98 (Natsal-3), 13 088 (EMIS), 752 (London-GMSHS) and 1119 (Scotland-GMSHS). The age-group distribution was similar between Natsal-3 and EMIS, but age differences remained between Natsal-3 and the other surveys (see online supplementary table S1). We found a reduction in the magnitude of many of the differences observed between Natsal-3 and EMIS when the samples were restricted to gay-identified MSM, although no formal test was used and results may be due to small sample sizes. As expected we found no difference in reporting female partner(s) (OR: 0.77, 95% CI 0.26 to 2.27); furthermore, UAI in EMIS changed to that of no significant difference as did same-sex anal sex in EMIS and Scotland-GMSHS (figure 1). However, differences still remained with MSM who identify as gay in convenience surveys reporting greater numbers of same-sex sexual partners, HIV testing and sexual health clinic attendance in the past year.

10.1136/sextrans-2015-052389.supp1 Supplementary table Demographic characteristics of MSM who identify as gay: Convenience surveys relative to Natsal-3

## Discussion

We estimated the proportion of MSM participating in Natsal-3 who report visiting gay venues and who searched for sexual partners online. The non-negligible proportion of MSM who did not do so illustrates the potential proportions of MSM who might be missed by convenience surveys that use venues and the internet for recruitment. However, it is important to recognise that MSM who do not report using the internet for seeking sex may still access gay-interest websites for other reasons. EMIS was promoted via a variety of websites, not all of which were dating sites, as well as being promoted via posters and postcards distributed at gay venues. It is estimated that 20% of participants were recruited via these other sources, for instance charities such as the Terrence Higgins Trust and GMFA (data not shown).

We then compared MSM participating in Britain's most recent national probability sample survey of sexual behaviour with participants in three major convenience surveys of MSM undertaken contemporaneously. Several sexual health indicators, specifically the number of male partners, anal sex, gonorrhoea diagnosis and HIV testing, were more commonly reported in the convenience surveys. This is most likely due to participants in Natsal-3 being recruited at home through a probability survey while convenience surveys are those of self-selected individuals in an environment where people typically look for a sexual partner. These differences remained in multivariable analyses, adjusting for sociodemographic differences between the surveys and Natsal-3. While greater similarity may exist between Natsal and convenience samples for MSM who identified as gay, some key differences remained.

There are several limitations to our study. The comparisons are predicated on the assumption that Natsal-3 provides an approximately representative sample of MSM. Natsal-3 achieved a response rate of 57.7%, in line with other major social surveys undertaken in Britain at the time,^12^ ^13^ but if those who did not participate systematically differed from those who did then Natsal-3 estimates will be biased. However, previous research suggests that, overall, Natsal-3 participants were demographically similar to participants in the 2011 UK census.^11^ With respect to sexual behaviour characteristics, research has found that participants taking part in Natsal-3 reported greater sexual risk behaviours compared with participants in a population-based general health survey,^14^ although, methodological differences exist that may, on balance, make Natsal-3's estimates more robust.

As a national probability sample survey, Natsal-3 has a relatively small sample size of MSM resulting in large CIs for rarer outcomes such as gonorrhoea diagnoses. Natsal-3's small sample size may also in part explain why fewer statistically significant differences were observed when we restricted the sample to MSM who identified as gay, although there was insufficient power to formally test an interaction. Due to Natsal-3's small sample size of MSM, we were unable to make geographically focused comparisons with London and Scotland. It is therefore uncertain to what extent differences in sexual health characteristics observed were due to selection bias in the venues or geographical differences.

We compared characteristics with similar question wording wherever possible. However, wording was not always identical, which may have affected our comparisons. For instance, men in Natsal-3 were asked a single question about how many men they had had sex with, whereas EMIS participants were asked separately about the number of their steady and non-steady sexual partners (table 1). It is possible therefore that combining responses to separate questions may result in a higher total number of partners than a single question.

Furthermore, the surveys used different data collection modes that may result in differences in reporting. However, many of the sexual health questions in Natsal were asked in the CASI, which is similar to EMIS.

This is the first study to compare data from MSM recruited to a national probability sample survey and MSM recruited to multiple major UK convenience surveys in an attempt to identify general rather than survey-specific differences. Such comparisons are needed on a regular basis to monitor whether differences exist, the magnitude of these differences and to identify possible reasons for them.^8^ The finding of greater reporting of sexual risk behaviours in convenience surveys than Natsal-3, which remain after adjusting for sociodemographic differences between the surveys, is consistent with previous studies.^6–8^ This suggests that men who are recruited to convenience surveys via gay-interest venues and websites continue to be different from MSM who do not. It is likely that data collected by such convenience surveys reflect a particular cross-section of MSM who are more likely to report greater risk behaviours, STI outcomes and HIV testing than the overall population of MSM and so most likely to benefit from health interventions.

A strength of online surveys is that they are able to collect data from a large sample and geographically broader target population more quickly and cheaply than venue-based surveys. This is a recruitment method that is continually growing in popularity, for instance, recruitment via social media and smartphone applications were recently used elsewhere.^15^ Furthermore, research has shown that participants in online surveys are less likely to report a gay identity, male-only partnerships and recent HIV testing than venue-based sampling,^16–20^ and as such there may be potential for estimates from online surveys of MSM to more accurately represent the heterogeneity of the whole MSM population than venue-based surveys. Online surveys also benefit from enabling participants to complete the questionnaire in an environment with greater anonymity, which may minimise social desirability bias compared with venue-based pen-and-paper questionnaire surveys,^21^ ^22^ although research of this benefit is inconclusive.^22^ Future research should examine the impact of different data collection methods for convenience surveys to ascertain, which results in the best data quality in terms of overall survey response, item non-response and prevalence estimates.

Applying adjustment weights based on demographic differences to convenience survey data could potentially account for some selection bias.^6^ However, the data presented here suggest that this may not be all that effective as adjusting for demographic differences between Natsal-3 and each individual convenience survey made little impact. In addition, weighting-up data from MSM who identify as bisexual or heterosexual in convenience surveys may not be statistically efficient due to their small number in these surveys.

Convenience samples also have the advantage that they can efficiently recruit MSM engaged in greater sexual risks who may be most likely to benefit from risk reduction interventions. They are also able to ask detailed questions about same-sex sexual behaviours and sexual health needs to inform the design and delivery of STI/HIV prevention interventions and policies. While these surveys are therefore essential, they do under-represent MSM less engaged in sexual risk behaviours and less engaged with sexual health services, who may have unmet sexual health needs. As MSM's use of the internet and other forms of communication technology develop, for example, apps, it is important that convenience surveys develop new ways of recruiting men, which reflect these changes in order to recruit representative (or where appropriate, targeted) samples.

To inform health service planning, it is important to triangulate the different sources of information. An example is the synthesis of multiple sources of data including Natsal and convenience surveys to estimate numbers of MSM with undiagnosed HIV in the whole MSM population.^23^ ^24^ However, further research is needed to develop triangulation methods and consider modifications to surveys so as to maximise the utility of data collected by probability and convenience surveys, providing added-value and strengthening the evidence-base for interventions that promote well-being in MSM.

**Key messages**

- Convenience surveys to date have tended to sample men in gay-orientated venues and so represent only a proportion of the men who have sex with men (MSM) population in Britain, who report greater risk behaviour.
- In contrast, probability sample surveys by definition are better placed to generate estimates representative of all MSM although based on smaller samples of MSM typically, and for a smaller number of behaviours.
- Differences between convenience and probability surveys reduce for some behaviours when focusing on MSM who identify as gay, but are not eliminated.
- As both sampling strategies have strengths and weaknesses, methods should be developed to triangulate data from probability and convenience surveys.
