# Supplementary material for: Men who have sex with men in Great Britain: comparing methods and estimates from probability and convenience sample surveys
Source: Sex Transm Infect. 2016 Mar 10;92(6):455–63. doi: 10.1136/sextrans-2015-052389 (PMC5013102; doi:10.1136/sextrans-2015-052389)
Supplement: Supplementary table — Demographic characteristics of MSM who identify as gay: Convenience surveys relative to Natsal-3 [file sextrans-2015-052389supp_table.pdf]

Men who have sex with men in Britain: comparison of estimates from a probability sample and community-based surveys

Supplementary Table S1: Demographic characteristics of MSM who identify as gay: Convenience surveys relative to Natsal-3

	Natsal-3	EMIS	London-GMSHS	Scotland-GMSHS
**Median age (IQR)**	34 (26 - 46)	36 (27 - 45)	33 (27 -40)	30 (24 - 40)
**Age group**				
18-24	17.0% (10.2% to 26.9%)	15.4%	12.5%	26.7%
25-34	34.3% (24.6% to 45.6%)	31.0%	43.0%	36.4%
25-44	18.7% (9.8% to 32.9%)	27.8%	31.0%	22.3%
45-64	30.0% (20.3% to 42.0%)	25.8%	13.6%	14.6%
P-value		0.450	0.002	0.014
**Academic qualifications**				
Degree level qualification	38.2% (27.1% to 50.6%)	48.7%		46.4%
Higher education, A-level or equivalent	24.7% (15.8% to 36.4%)	33.3%		37.4%
GCSE, O-Level or equivalent	29.1% (20.2% to 39.9%)	15.6%		14.6%
None	8.1% (3.6% to 17.0%)	2.5%		1.5%
P-value		<0.001		<0.001
**Employment**				
Employed	66.7% (55.6% to 76.3%)	67.6%	88.3%	71.5%
Other/Unemployed	33.3% (23.7% to 44.4%)	32.4%	11.7%	28.5%
P-value		0.872	<0.001	0.291
**Ethnicity (binary)**				
White	98.3% (88.9% to 99.8%)		84.4%	
Non-white	1.7% (0.2% to 11.1%)		15.6%	
P-value			0.004	
**London resident**				
No	75.1% (61.3% - 85.1)	60.4%		
Yes	24.9% (14.9-38.7)	39.6%		
P-value		0.035		
**Urban area**				
Rural or Town area (<10,000)	11.0% (6.0% to 19.3%)	8.8%	17.2%	9.5%
Urban area (>10,000)	89.0% (80.7% to 94.0%)	91.2%	82.8%	90.5%
P-value		0.467		0.126
**Country**				
England	85.0% (74.8% to 91.6%)	90.1%		
Scotland	7.4% (3.6% to 14.7%)	6.9%		
Wales	7.5% (3.0% to 17.6%)	3.0%		
P-value		0.075		
**Attraction scale**				
Opposite sex only	0% ()	0.0%		
More often opposite sex, and at least once same sex	0.6% (0.1% to 3.9%)	0.0%		
About equally often to opposite sex and same sex	0.9% (0.1% to 5.9%)	0.1%		
More often same sex, and at least once opposite sex	42.8% (31.8% to 54.5%)	10.9%		
Same sex only	55.8% (44.1% to 66.9%)	89.0%		
P-value		<0.001		
Denominator	98, 93	13088	752	1119

p values: chi^2 test compared to Natsal-3
